# Changes in Physicochemical Properties, Volatile Flavor Compounds and Microbiota in *Phascolosoma esculenta* Muscle During Frozen Storage at −20 °C

**DOI:** 10.3390/biology15191789

**Published:** 2026-10-08

**Authors:** Binxin Cai, Fengfang Zhou, Qi Wei, Ding Li, Weiqing Huang

**Affiliations:** 1 College of Marine Sciences, Ningde Normal University, Ningde 352100, China; 2 College of Biological Science and Engineering, Ningde Normal University, Ningde 352100, China

**Keywords:** *Phascolosoma esculenta*, frozen storage, volatile flavor compounds, microbiota composition, metabolites, protein degradation, lipid oxidation

## Abstract

*Phascolosoma esculenta* is a valuable marine seafood, which suffers quality and flavor deterioration with the extension of frozen storage time. To reveal the degradation mechanism, physicochemical properties, microbiota, metabolites, and volatiles were investigated in *P. esculenta* muscle during frozen storage (−20 °C). Water-holding capacity (WHC) decreased to 77.87%, while the content of total volatile basic nitrogen (TVB-N) and thiobarbituric acid reactive substances (TBARSs) increased to 18.85 mg/100 g and 1.18 mg MDA/kg, respectively. Long-term frozen storage reduced microbial diversity. *Macrococcus* and *Nitrospira* became the predominant genera after 360 days of frozen storage, and their predominance was associated with muscle flavor deterioration. Untargeted metabolomics identified 4299 metabolites. Differential metabolites were mainly involved in purine, nucleotide, arachidonic acid, and amino acid metabolism. Volatile profiles were markedly altered during frozen storage. Freshness-related aldehydes decreased markedly, whereas off-flavor alcohols (e.g., 1-octen-3-ol) and sulfur-containing volatiles (e.g., dimethyl trisulfide) continuously accumulated. Pearson correlation analysis showed that physicochemical spoilage indicators, cold-adapted microorganisms, and differential lipid metabolites were closely associated with the generation of undesirable off-odors. This study reveals the interactive effects of protein degradation, lipid oxidation, and microbial succession during frozen storage.

## Simple Summary

*Phascolosoma esculenta* is a valuable marine seafood, which suffers quality and flavor deterioration with the extension of frozen storage time. This study investigated changes in its muscle during storage at −20 °C. Water-holding capacity (WHC) decreased, while the content of total volatile basic nitrogen (TVB-N) and thiobarbituric acid reactive substances (TBARSs) increased. Microbial diversity declined, with *Macrococcus* and *Nitrospira* becoming predominant after 360 days. Metabolomics and volatile analysis revealed altered purine, nucleotide, arachidonic acid, and amino acid metabolism, together with accumulation of off-flavor compounds. These changes were closely associated with protein degradation, lipid oxidation, and microbial succession.

## 1. Introduction

*Phascolosoma esculenta* (*P. esculenta*) is a marine invertebrate of high nutritional and economic value, endemic to intertidal mudflats along the coast of Southeast China [1]. Well-known for its delicious taste and high protein content, *P. esculenta* has long been prized as a high-quality seafood ingredient and a traditional nutritional tonic [2,3]. However, after capture, fresh *P. esculenta* undergoes quality deterioration caused by changes in body texture properties, endogenous protease activity, water activity, and other indicators [4]. This significantly reduces its commercial value. Therefore, proper preservation and processing are critical for the development of the *P. esculenta* industry.

At present, both unprocessed live/fresh products and processed products have a short shelf life. Freezing is widely recognized as the most efficient and commonly used technique for prolonging the shelf life of aquatic products [5]. Nevertheless, the specific mechanisms governing quality and flavor deterioration in *P. esculenta* during frozen storage remain inadequately elucidated. Although subzero temperatures can substantially inhibit microbial proliferation and reduce enzymatic activity, psychrotrophic microorganisms and endogenous enzymes are not completely inactivated and still retain residual metabolic activity during extended storage periods [6]. These sustained biochemical reactions gradually degrade muscle integrity via protein denaturation, lipid oxidation, and the subsequent formation of undesirable off-flavors [7,8]. Therefore, developing a comprehensive understanding of these deterioration pathways is essential for optimizing cryopreservation protocols and improving the final product quality of frozen *P. esculenta*.

Flavor is a critical quality attribute of aquatic food products. Volatile organic compounds (VOCs) serve as an indirect indicator for evaluating freshness and quality [9]. HS-SPME-GC-MS is widely used in food flavor research [10]. Due to its advantages of high throughput and comprehensive coverage, LC-MS has been widely employed for metabolite identification during food spoilage [11]. Notably, multi-omics approaches have proven valuable for elucidating the dynamic quality changes that occur in various aquatic products under frozen storage. Guo et al. [7] revealed the multi-factor synergistic molecular network underlying the quality deterioration of frozen-stored crab meat via the integration of volatilomics and microbiomics analyses. The dynamic interplay of physicochemical properties, volatile flavor compounds, and microflora changes in the muscles of swimming crab during frozen storage was investigated by Du et al. [12]. LC-MS-based untargeted metabolomics approach was applied to profile metabolite variations in sea urchin under different packaging conditions during frozen storage [13]. However, systematic investigations that employ these integrated strategies to characterize changes in the flavor and quality of frozen *P. esculenta* remain limited.

This study integrated physicochemical indicators, volatile flavor profiling, microbiome sequencing, and untargeted metabolomics to investigate quality changes in *P. esculenta* muscle over 360 days of frozen storage, with special emphasis on the interplay among storage time, VOCs, and key spoilage metabolite accumulation.

## 2. Materials and Methods

### 2.1. Sample Preparation and Storage Conditions

Fresh *P. esculenta* (individual weight: 4.2 ± 0.34 g) were collected from a single provider at a local market in Ningde City, China. A 5 kg samples of *P. esculenta* were immediately eviscerated and thoroughly washed with pre-cooled sterile water (4 °C) to obtain body wall muscle. The cleaned muscle samples were packaged in sterile polyethylene bags (approximately 100 g per bag) and stored at −20 °C. The storage period lasted for 360 days, with sampling conducted on days 0 (fresh *P. esculenta*), 90, 180, 270, and 360 for various analyses.

### 2.2. Determination of WHC

WHC was determined according to the method reported by Chen et al. [14], with slight modifications. 5 g of *P. esculenta* muscle sample was accurately weighed (recorded as W_1_, the weight before centrifugation), wrapped in double-layer qualitative filter paper and centrifuged at 4000 r/min for 10 min at 4 °C (Centrifuge 3-16KL, Sigma, Osterode am Harz, Germany). After centrifugation, the free water on the sample surface was gently blotted dry with filter paper, and the sample was weighed again (recorded as W_2_, the weight after centrifugation). The WHC was calculated using the following formula:

WHC (%) = (W_2_/W_1_) × 100

### 2.3. Determination of pH

Following the standard GB 5009.237-2016 [15], pH was measured on muscle samples. Briefly, 4.00 g of sample was homogenized with 40 mL of pre-cooled distilled water at 10,000 r/min for 1 min (IKA T18, IKA-Werke, Staufen, Germany). Then the homogenate was centrifuged (5000 r/min, 5 min, 4 °C). The supernatant was isolated, and its pH value was read using a digital pH meter (FE28, Mettler Toledo, Greifensee, Switzerland).

### 2.4. Determination of TVB-N Content

TVB-N content was quantified according to GB 5009.228-2016 [16]. Briefly, samples (5.0 g) were extracted with 25 mL of water under shaking for 30 min, then filtered. Then, 1.0 mL of filtrate was distilled on a diffusion plate. The ammonia vapor was captured by 1 mL of boric acid (20 g/L), after which the solution was titrated with 0.01 mol/L HCl. TVB-N content was ultimately expressed as mg/100 g.

### 2.5. Determination of TBARS Content

TBARS content was assayed per GB 5009.181-2016 [17]. Briefly, 5.0 g of muscle sample was mixed with 25 mL of 7.5% trichloroacetic acid solution, shaken for 30 min, and filtered. Next, 5.0 mL of the clear filtrate was combined with 5.0 mL of thiobarbituric acid solution (0.02 mol/L) and placed in a 90 °C water bath for 30 min. The absorbance was finally determined (Thermo Fisher Scientific, Waltham, MA, USA). The absorbance of the MDA standard series solutions was measured, and a standard curve was plotted, yielding the regression equation: Y = 0.345X + 0.006 (R^2^ = 0.994). The MDA content was calculated using the following equation:

X = c × V × 1000/(m × 1000).

where X—MDA content in the sample (mg/kg); c—MDA concentration of the standard series solution (μg/mL); V—final volume of the sample solution (mL); m—sample mass (g); 1000—conversion factor.

### 2.6. Bacterial Diversity Analysis

Nine samples were subjected to bacterial community profiling. DNA extraction was carried out using the E.Z.N.A.^®^Soil DNA Kit (Omega Bio-tek, Norcross, GA, USA). The V3–V4 region of the 16S rRNA gene was amplified with primers 338F and 806R. The thermocycling conditions were set as: initial denaturation at 95 °C for 3 min; 27 cycles at 95 °C (30 s), 55 °C (30 s), and 72 °C (30 s); followed by a final extension at 72 °C for 10 min. Paired-end sequencing of the amplicons was performed on the Illumina NextSeq 2000 platform. Sequence reads were clustered into OTUs at 97% similarity via Usearch. Species annotation was performed against the Silva 16S rRNA database (v138) to analyze bacterial community composition, abundance, and diversity.

### 2.7. Untargeted Metabolomics Analysis

Eighteen samples were analyzed via untargeted metabolomics. Briefly, 50 mg of *P. esculenta* muscle was mixed with 400 μL of methanol/water (4:1, *v*/*v*) containing four internal standards (0.02 mg/mL L-2-chlorophenylalanine, etc.) in a 2 mL grinding tube and ultrasonicated at low temperature for 30 min. After centrifugation, the supernatant was collected for LC-MS/MS analysis on a UHPLC-Orbitrap Exploris 240 system. Metabolites were separated on a Waters ACQUITY UPLC HSS T3 column (100 mm × 2.1 mm i.d., 1.8 μm) with mobile phase A (water/acetonitrile, 95:5, *v*/*v*, containing 0.1% formic acid) and B (acetonitrile with 0.1% formic acid). The gradient was: 0–1.5 min, 5% B; 1.5–2 min, 5–10% B; 2–4.5 min, 10–30% B; 4.5–5 min, 30–100% B; 5.5–6.3 min, 100% B. Flow rate was 0.40 mL/min, column temperature was 40 °C, and injection volume was 5 μL. MS acquisition used positive mode (+3400 V) and negative mode (−3000 V), with source temperature 350 °C and sheath/auxiliary gas flows of 60/20 arb. A pooled quality control sample was prepared by mixing equal volumes of all samples and processed identically.

Raw LC-MS data were imported into Progenesis QI (Version 2.0, Waters, Milford, MA, USA) for baseline filtering, peak detection and related processing. Metabolites were annotated by matching MS/MS spectra against HMDB, Metlin, etc. Missing values were removed following the 80% rule, and remaining gaps were filled with the minimum values of the original matrix. Data were then total-sum normalized, filtered to remove variables with QC RSD > 30%, and log10 transformed. PCA and OPLS-DA were performed using the R package “ropls” (Version 1.6.2). Differential metabolites were screened by VIP > 1 (OPLS-DA) and Student’s *t*-test *p* < 0.05.

### 2.8. Analysis of VOCs

The analysis of volatile flavor compounds was performed via SPME-GC-MS. Briefly, 3 g of sample was weighed into a 20 mL headspace vial, containing 2 µL of internal standard (n-Pentadecane-d32) and 5 mL of saturated NaCl, and then crimp-sealed. After thermal equilibration at 80 °C for 20 min, a pre-conditioned DVB/Carbon WR/PDMS fiber (120 µm × 20 mm; Thermo Fisher) was introduced into the vial headspace for 10 min analyte adsorption. The fiber was then retracted and injected into the GC-MS port (split mode), with 1 µL of sample solution. Separation was accomplished on a VF-WAXms column (25 m × 0.25 mm × 0.2 µm) using 99.999% helium carrier gas at 1 mL/min constant flow, with the split/splitless injector at 240 °C. The temperature gradient commenced at 40 °C, rising to 120 °C at 8 °C/min and subsequently to 230 °C at 20 °C/min, with a 4.5 min final isothermal hold. The MS operated in electron ionization mode (70 eV) with the source at 250 °C, scanning the *m*/*z* 35–500 range in full-scan mode. VOCs were identified by matching mass spectra and retention indices to those in the NIST-2023 library, the Thermo Scientific GC-Orbitrap Flavor and Fragrances v1.0 library, and an in-house Majorbio database. Three criteria were applied to ensure reliable identification beyond NIST spectral matching alone: (i) a high-resolution filtering (HRF) score > 80 obtained using Compound Discoverer software (version 3.3); (ii) a match factor based on the MS pattern (SI) > 600; and (iii) a retention index deviation (ΔRI) < 50.

### 2.9. Statistical Analysis

All determinations were expressed as means ± SD. Six biological replicates were used for physicochemical indices (pH, WHC, TBARS, TVB-N) and non-targeted metabolomics analysis, while three biological replicates were adopted for bacterial diversity analysis and VOC detection. No technical replicates were performed for any assay. Data analysis was performed using SPSS 20.0. One-way ANOVA was applied to compare differences among different storage time points. Significance was accepted at *p* < 0.05. Origin 2022 was used for plotting.

## 3. Result

### 3.1. Changes in WHC

**Figure 1:**
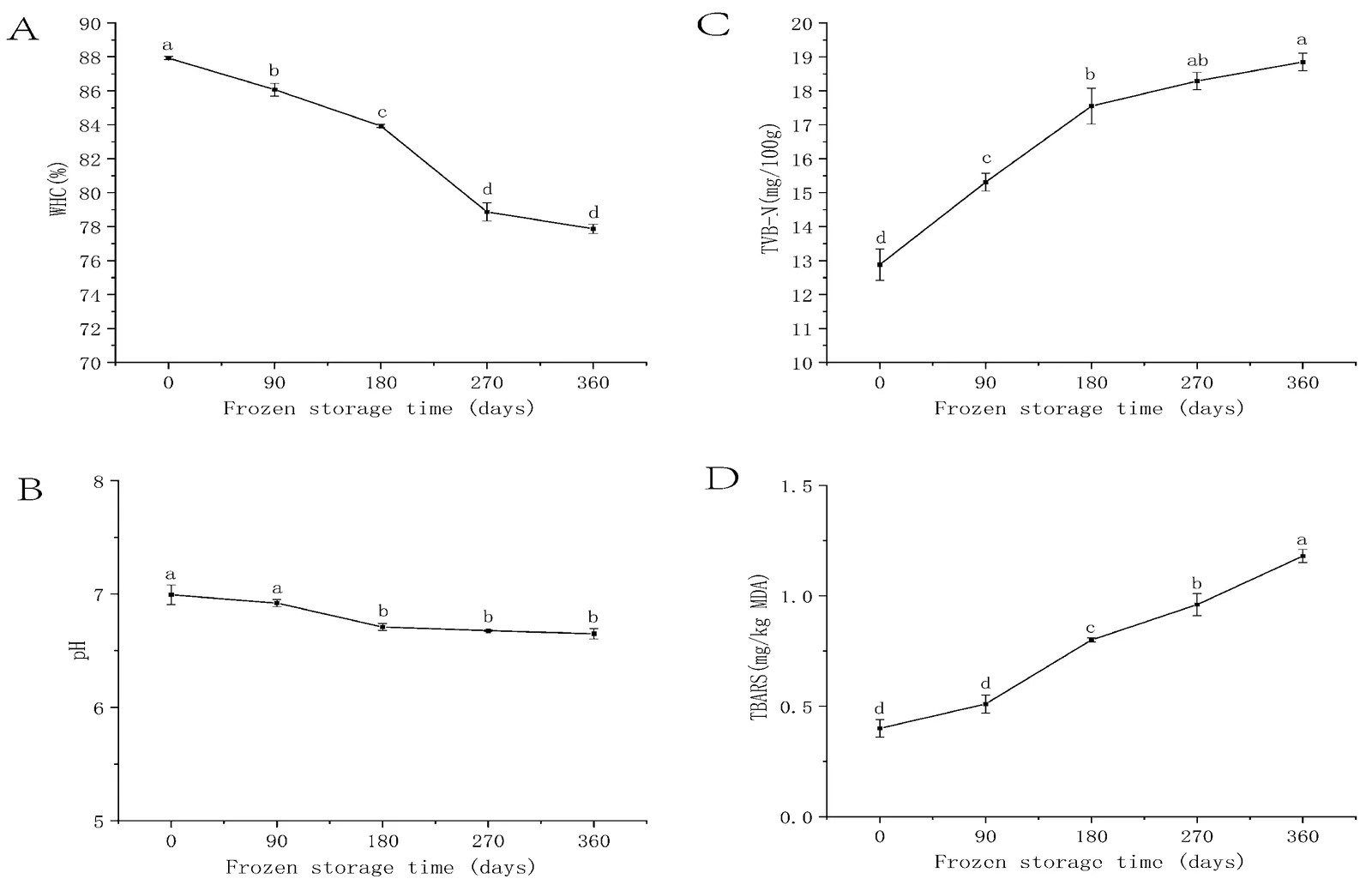
Changes in physicochemical quality indicators of *P. esculenta* muscle during frozen storage. (**A**) WHC; (**B**) pH; (**C**) TVB-N content; (**D**) TBARS content. Note: Different lowercase letters indicate significant differences between different freezing times (*p* < 0.05).

WHC is a critical indicator to assess the water-binding capacity of muscle proteins. Throughout the 360 d frozen storage period, the WHC of *P. esculenta* muscle exhibited a continuous declining trend (Figure 1A). The initial WHC of fresh samples (0 d) reached 87.93%, which slowly declined to 83.93% at day 180. A marked reduction was observed between day 180 and day 270, followed by a slight decline in the later stage.

### 3.2. Changes in pH Content

The pH is a vital indicator of freshness in aquatic products. During the storage period, the pH values of *P. esculenta* muscle exhibited a trend of slight decrease (Figure 1B). During the early storage phase (days 0–90), pH content showed a declining trend, dropping to 6.92 by day 90. The pH content was significantly reduced by day 180 (*p* < 0.05) and remained stable thereafter.

### 3.3. Changes in TVB-N Content

TVB-N, a critical chemical indicator of alkaline nitrogenous spoilage products such as biogenic amines, trimethylamine, dimethylamine, etc., is widely used to evaluate the degree of spoilage in aquatic products. As shown in Figure 1C, the TVB-N content in fresh muscle was initially 12.88 mg/100 g and increased continuously throughout the storage period. A rapid elevation was observed within the first 0–180 days, reaching 17.55 mg/100 g at day 180. The index subsequently increased moderately and reached 18.85 mg/100 g at day 360, which remained far below the statutory limit of 30 mg/100 g for marine aquatic products specified in GB 2733-2015 [18]. These results reveal that frozen preservation can effectively retard protein degradation and the accumulation of alkaline nitrogenous substances.

### 3.4. Changes in TBARS Content

TBARS content, a reliable indicator of lipid oxidation extent in muscle tissue, was measured as MDA levels. As illustrated in Figure 1D, the TBARS content of *P. esculenta* muscle progressively increased with extended storage time. During the early storage period (days 0–90), TBARS content increased slowly. From day 90 onward, the TBARS content accelerated significantly, increasing to 1.18 mg MDA/kg at the end of storage (day 360). These findings indicated that lipid oxidation became more severe with the extension of frozen storage duration.

### 3.5. Bacterial Diversity

**Figure 2:**
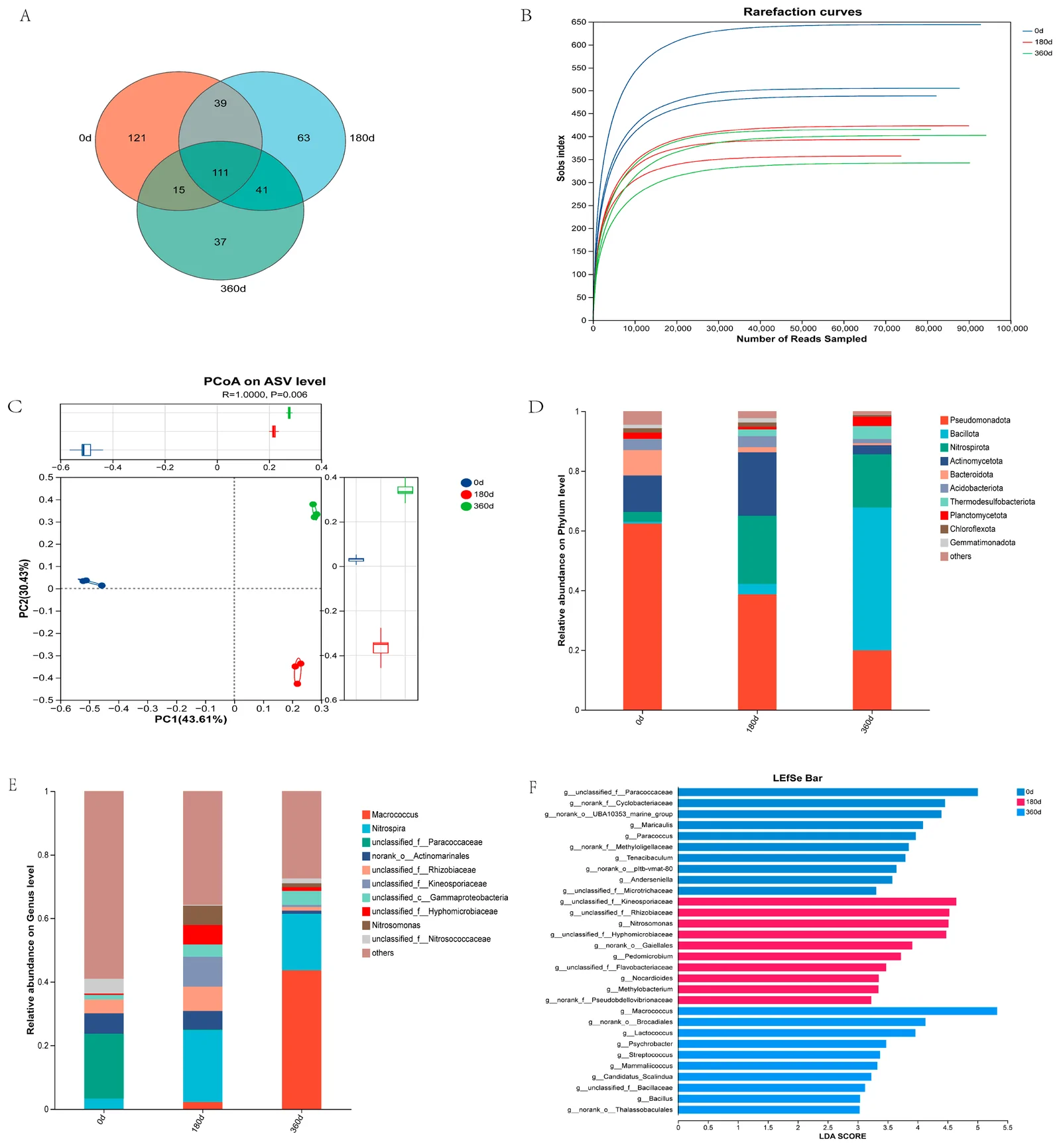
Bacterial community composition and diversity of *P. esculenta* muscle during frozen storage. (**A**) Venn analysis of species; (**B**) the dilution curve based on the average Sobs index; (**C**) PCoA at the OTU level; (**D**) Microbial community at the phylum level; (**E**) Microbial community at the genus level; (**F**) LDA value of differential species.

Microbial communities in nine *P. esculenta* muscle samples were characterized via 16S rRNA high-throughput sequencing. After stringent read filtering, 969,567 clean sequences were retained (Table S1). Clustering at 97% nucleotide identity identified 427 OTUs across all groups. The 0 d, 180 d, and 360 d groups harbored 121, 63, and 37 unique OTUs, respectively. As storage time progressed, the OTU count per sample declined steadily, reflecting a negative impact of freezing on microbial richness (Figure 2A). The Sobs-based rarefaction curves (Figure 2B) reached saturation for all groups, ensuring sufficient sequencing depth for downstream comparisons. The dilution curves of the 180 d and 360 d groups showed similar trends, whereas the curve of the 0 d group was clearly separated from them, indicating that frozen storage altered the microbial community diversity level in the muscle of *P. esculenta*.

Alpha diversity analysis showed that frozen storage significantly altered the species richness and evenness of the microbial community in the muscle of *P. esculenta* (Table 1). The Chao1 and Ace indices were significantly reduced in the 180 d and 360 d frozen storage groups, confirming that long-term low-temperature storage caused a decrease in microbial community species richness. The Shannon and Simpson indices indicated that the microbial diversity in the 360 d group was significantly lower than in the other two groups. This finding further demonstrated that low-temperature storage reduced the evenness of the microbial community, and the community gradually shifted toward the enrichment of several dominant species.

Beta diversity analysis further confirmed significant differences in the microbial community structure of *P. esculenta* muscle among different frozen storage durations (Figure 2C). The phylum-level species composition is presented in Figure 2D. In the 0 d group, the dominant phylum was Pseudomonadota (62.38%), which exhibited the highest relative abundance. As storage time prolonged, its proportion gradually decreased, dropping to 19.90% after 360 days of frozen storage. In contrast, the relative abundance of Bacillota showed a significant upward trend during storage, rising from 0.64% at 0 d to 47.85% at 360 d. Meanwhile, after 180 days of frozen storage, the relative abundances of Nitrospirota and Actinomycetota both increased substantially and then declined; Over the storage period, the relative abundance of Bacteroidota decreased markedly. The results indicated that the long-term storage process induced significant succession in bacterial community structure at the phylum level, accompanied by a marked shift in the dominant bacterial phyla, reflecting the differential adaptive capabilities of various microbial groups to storage environmental conditions. Figure 2E showed that the proportion of *Macrococcus* exhibited a significant upward trend, gradually increasing from a very low abundance at day 0 to 43.62% at day 360, becoming the dominant genus. The relative abundance of *Nitrospira* increased continuously with prolonged storage time, indicating its strong adaptability to long-term preservation conditions.

LEfSe analysis identified 30 microbial taxa with significant linear discriminant analysis (LDA) scores (Figure 2F). At the initial storage stage (0 d), the microbial community was predominantly composed of unclassified *Paracoccaceae*, norank *Cyclobacteriaceae*, norank *UBA10353* marine group, and *Maricaulis*, reflecting the natural microbiota associated with the marine habitat. After 180 days of frozen storage, the community was enriched in *Nitrosomonas*, *Kineosporiaceae*, *Rhizobiaceae*, and *Hyphomicrobiaceae*, suggesting a transitional stage driven by nitrogen released from protein degradation. By day 360, *Macrococcus* and norank *Brocadiales* emerged as the dominant biomarkers, indicating that prolonged frozen storage selected for stress-tolerant and anaerobic ammonium-oxidizing bacteria.

### 3.6. Differential Metabolites Analysis

**Figure 3:**
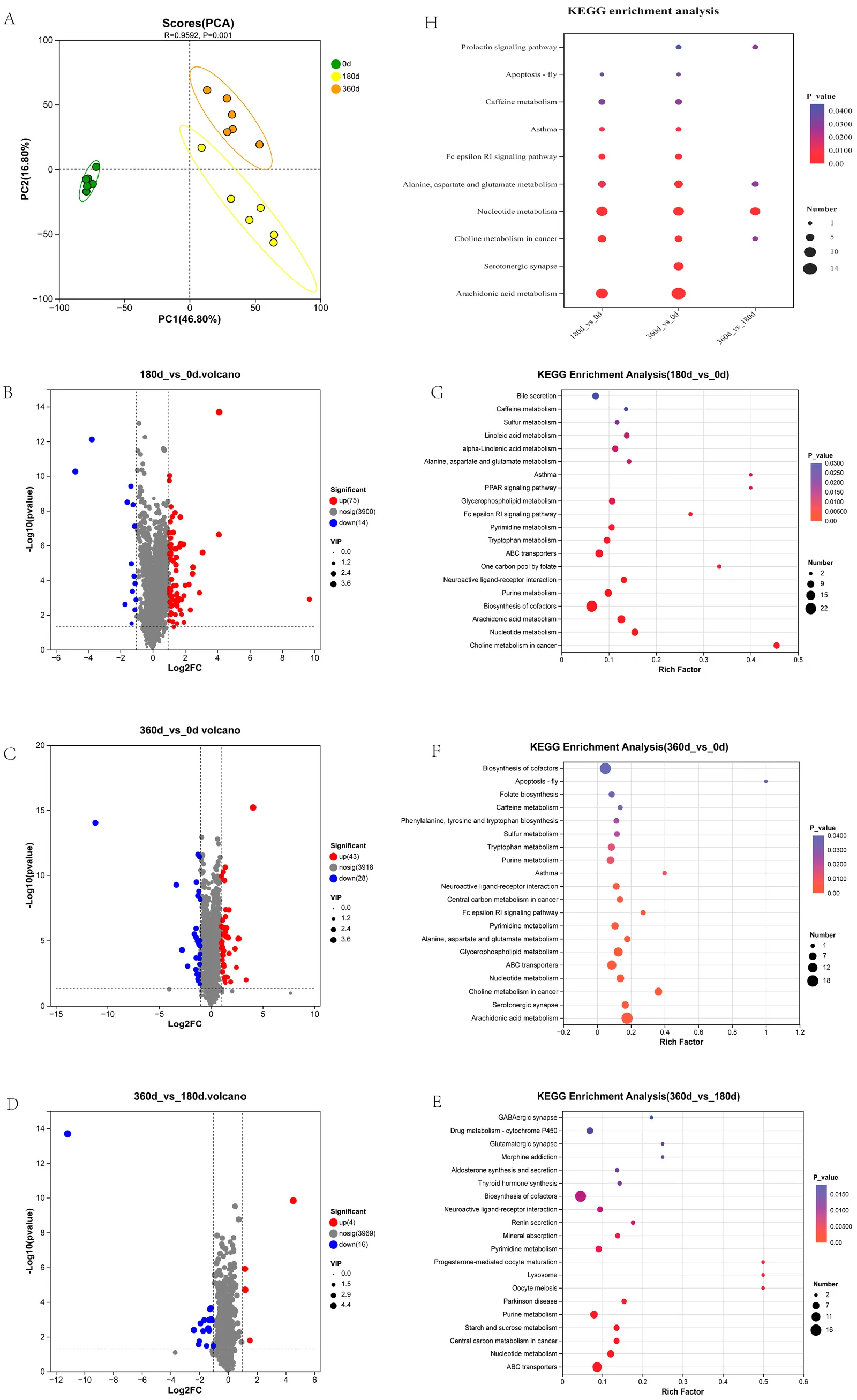
Differences in metabolites of *P. esculenta* muscle during frozen storage. (**A**) PCA of metabolomic data in different storage times; (**B**) Volcano plot of different metabolites of fresh and frozen storage 180 days, (**C**) fresh and frozen storage 360 days, (**D**) frozen storage 180 days and 360 days; (**E**) KEGG pathway analyses of frozen storage 180 days and 360 days, (**F**) fresh and frozen storage 180 days, (**G**) frozen storage 360 days; (**H**) KEGG pathway analysis of pairwise comparisons.

Untargeted metabolomics was used to demonstrate the metabolite changes in meat during frozen storage. Through metabolomics analysis, a total of 4299 metabolites were detected in fresh and frozen muscle samples (Table S2). The PCA plot showed obvious separations in the metabolites of *P. esculenta* muscle across the 0 d, 180 d and 360 d frozen-storage groups (Figure 3A). Based on the screening criteria (Log_2_ (FC) > 1, *p* < 0.05, and VIP > 1), differential metabolites were identified. There were 89 differential metabolites between the 0 d and 180 d groups, of which 75 metabolites were upregulated and 14 were downregulated (Figure 3B). The upregulated metabolites included (S)-Oleuropeic Acid, Gly-Phe-Ile, and P-Coumaryl Alcohol, while the downregulated metabolites included Thr-Leu-Asp-Glu and N-Acetylprocainamide. These differential metabolites were mainly enriched in nucleotide metabolism, arachidonic acid metabolism, purine metabolism, tryptophan metabolism, pyrimidine metabolism, and biosynthesis of cofactors (Figure 3G). Compared with the 0-day group, the 360-day group had 43 differential metabolites increased and 28 decreased (Figure 3C).

Differential metabolites were mainly enriched in arachidonic acid metabolism, nucleotide metabolism, alanine, aspartate and glutamate metabolism, pyrimidine metabolism, and glycerophospholipid metabolism (Figure 3F). In the 180 day vs. 360 day comparison, 4 metabolites were upregulated, including Thr-Leu-Asp-Glu, 4-Hydroxycinnamoylagmatine, Asp-Asp-Met and 16 metabolites were downregulated, including Tetrahydroharman-3-Carboxylic Acid and Carvedilol (Figure 3D). KEGG pathway analysis revealed that the differential metabolites between the 180 d and 360 d group were mainly enriched in pathways related to ABC transporters, nucleotide metabolism, pyrimidine metabolism, and purine metabolism (Figure 3E). Furthermore, KEGG pathway enrichment analysis showed that the differential metabolites were mainly enriched in arachidonic acid metabolism, nucleotide metabolism, and amino acid metabolism (including alanine, aspartate, and glutamate pathways) between the fresh group and the frozen storage groups (180 d and 360-d). The differential metabolites were mainly enriched in nucleotide metabolism between the 180 d and 360 d frozen groups (Figure 3H).

### 3.7. VOC Analysis

**Figure 4:**
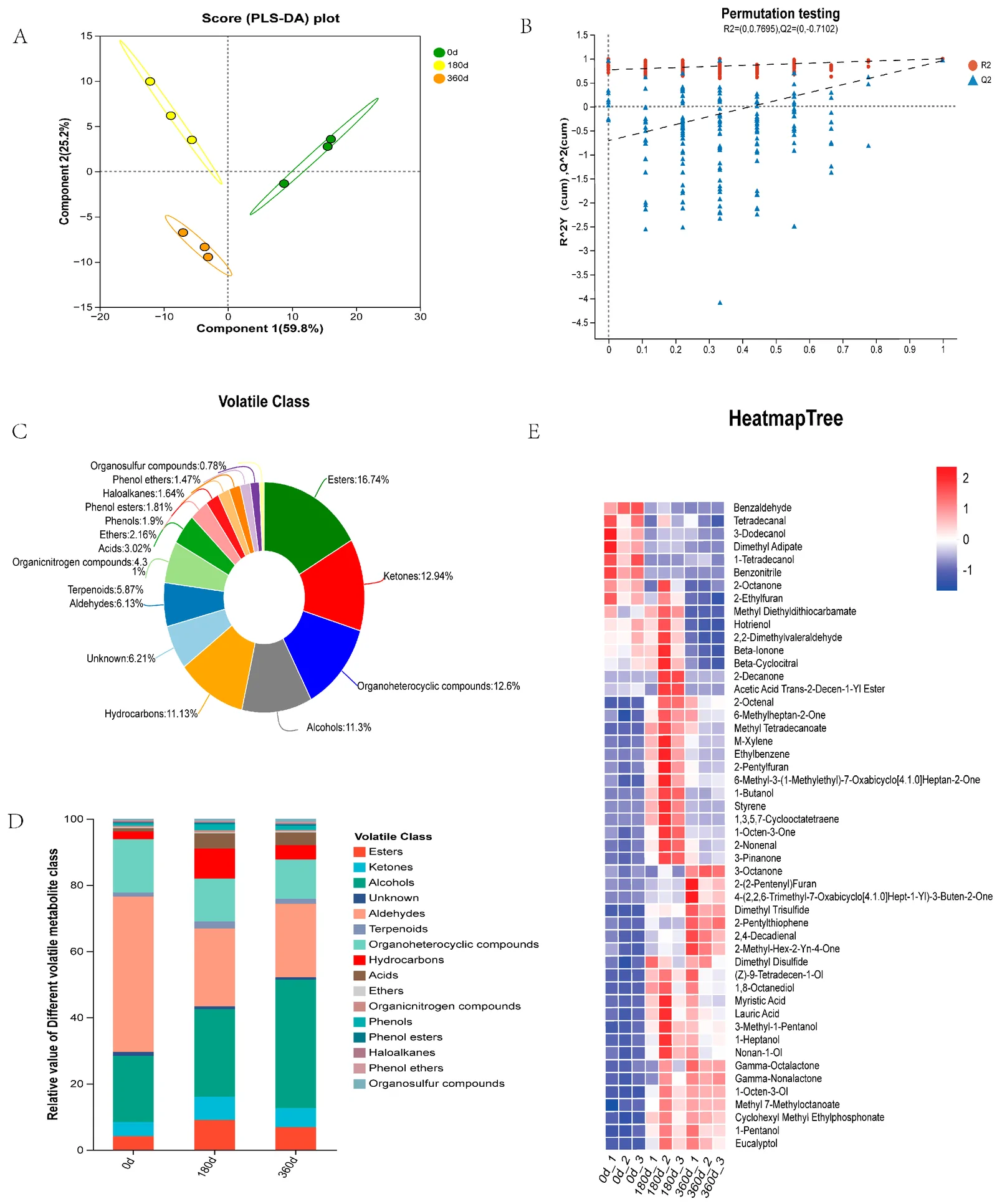
VOC changes of *P. esculenta* muscle during frozen storage. (**A**) OPLS-DA score plots; (**B**) Permutation tests; (**C**) the classification proportion ring chart of the identified total volatile substances; (**D**) relative content accumulation chart of the identified total volatile substances; (**E**) clustering heatmap of VOCs.

VOCs could serve as indicators of the *P. esculenta* muscle odor and quality during frozen storage. PCA of the VOCs clearly separated the three groups, indicating distinct differentiation (Figure S1). To enhance VOC-based discrimination among *P. esculenta* samples and reduce noise interference, we performed partial least squares discriminant analysis (PLS-DA) (Figure 4A). The model demonstrated robust stability and reliable predictive performance, with no evidence of overfitting (Figure 4B). The VOC profiles of *P. esculenta* samples are summarized in Figure 4C. A total of 1157 VOCs were detected, including esters (194, 16.74%), ketones (150, 12.94%), alcohols (131, 11.3%), aldehydes (71, 6.13%), and others. The relative contents of VOCs in *P. esculenta* muscle at three storage time points were shown in Figure 4D and Table S3.

Aldehydes accounted for the highest proportion (46.86%) in the 0 d group and were the main contributors to the flavor of fresh *P. esculenta* muscle. The relative aldehyde content decreased dramatically with extended frozen storage, falling to 23.44% and 22.19% in the 180 d and 360 d. This suggested that prolonged freezing caused substantial aldehyde degradation or conversion, resulting in a marked loss of fresh flavor characteristics. Conversely, the relative content of alcohols accumulated progressively, rising significantly from 20.90% in the 0 d to 26.43% in the 180 d and peaking at 38.96% in the 360 d. At this stage, alcohols became the dominant volatile category, implying that carbonyl reduction was promoted during prolonged freezing. Esters and ketones both increased initially and decreased afterwards, indicating a transient enhancement of esterification and oxidation at the early freezing stage, followed by a decrease as substrates were consumed or transformed later on. In conclusion, frozen storage progressively shifted the volatile flavor profile of muscle from aldehyde-dominated fresh notes to oxidative deterioration characterized by alcohol accumulation. The persistent decline in aldehydes, along with marked increases in alcohols and acids, were the primary factors driving this flavor deterioration.

To more intuitively observe the changes in VOCs in the samples, we selected the VOCs with the top 50 abundances for cluster analysis (Figure 4E). Putrid flavor markers including dimethyl trisulfide, 1-octen-3-ol, and 2, 4-decadienal, exhibited a sustained accumulation pattern throughout the storage period. Notably, dimethyl trisulfide, a typical spoilage biomarker for aquatic products, exhibited a dramatic fold increase at day 360 relative to fresh samples, which strongly demonstrates progressive microbial deterioration over prolonged frozen storage. In contrast, fresh-associated aroma compounds, particularly benzaldehyde and beta-ionone, decreased markedly during storage.

### 3.8. Correlation Analysis

**Figure 5:**
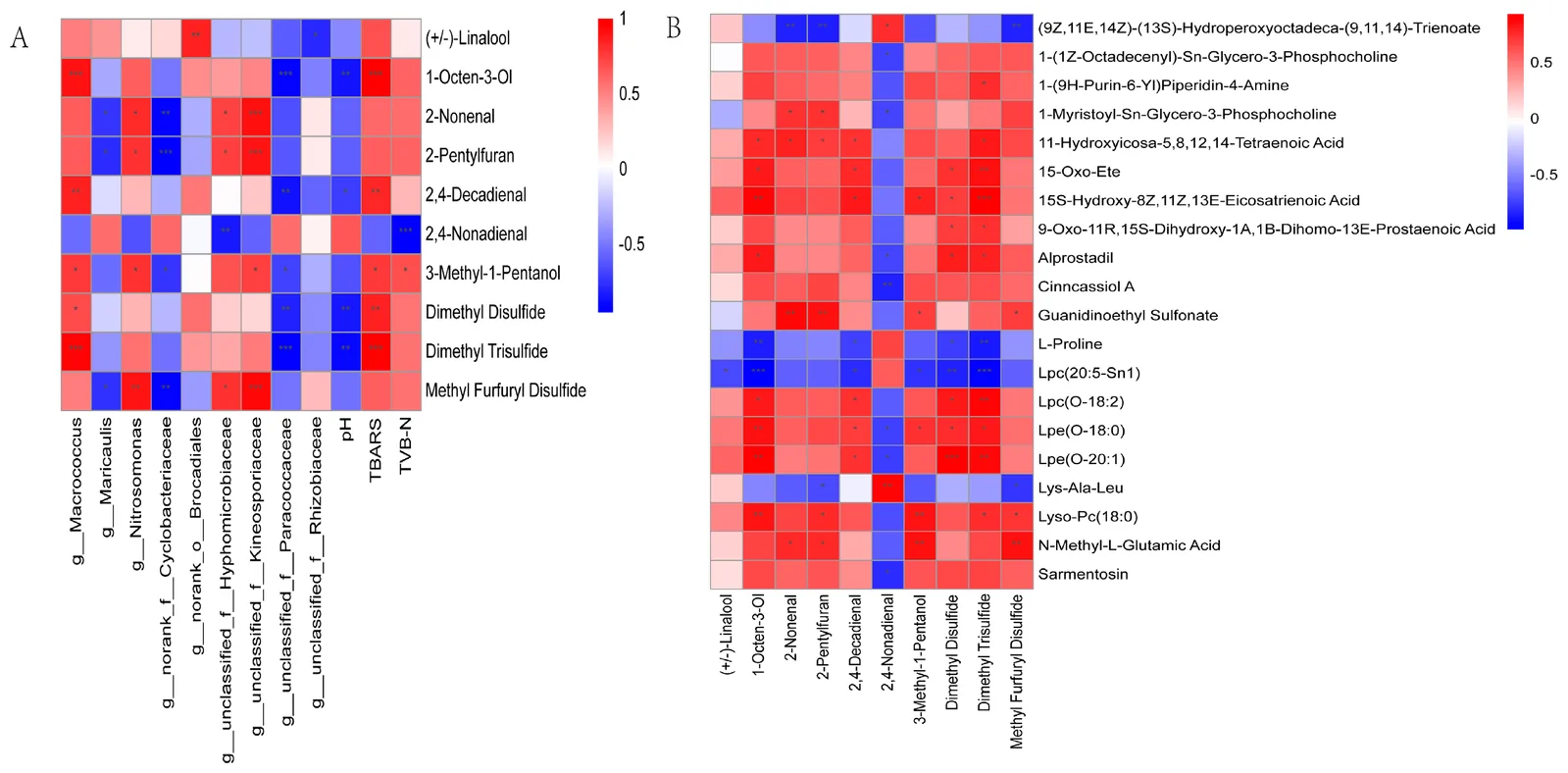
The correlations of physicochemical properties, microbiota, metabolites, and flavor indicators of *P. esculenta* muscle during frozen storage. (**A**) Pearson correlation analysis of basic physicochemical indicators, main microorganisms, and key volatile flavors; (**B**) the correlation analysis of key volatile flavors and key differential metabolite. Note: * *p* < 0.05, ** *p* < 0.01, *** *p* < 0.001.

In Figure 5A, the correlation analysis revealed that TBARSs exhibited a significant positive correlation with 1-octen-3-ol, dimethyl trisulfide, and dimethyl disulfide, suggesting that the degree of lipid oxidation is highly correlated with the accumulation of VOCs. The pH value showed a significant negative correlation with dimethyl trisulfide, 1-octen-3-ol, and dimethyl disulfide, indicating that an acidic environment may inhibit the formation of these compounds or promote their degradation.

TVB-N exhibited an extremely significant negative correlation with 2,4-nonadienal and a significant positive correlation with 3-methyl-1-pentanol. These correlations suggested an association between protein degradation products and the formation of specific aldehydes and alcohols. Among microbial genera, *Macrococcus* showed a significant positive correlation with 1-octen-3-ol, 2,4-decadienal, 3-methyl-1-pentanol, dimethyl disulfide, and dimethyl trisulfide. In contrast, unclassified f_*Paracoccaceae* exhibited a significant negative correlation with most of the aforementioned compounds, with the correlations being particularly pronounced for 1-octen-3-ol, and dimethyl trisulfide. In addition, unclassified f_*Kineosporiaceae* displayed an extremely strong positive correlation with methyl furfuryl disulfide, 2-pentylfuran and 2-nonenal, whereas norank f_*Cyclobacteriaceae* demonstrated a strong negative correlation with these three flavor compounds. The results indicated that lipid oxidation, protein degradation, and microbial activity were the primary causes of the deterioration of the quality and flavor of *P. esculenta*.

In Figure 5B, key off-odor volatiles such as dimethyl disulfide, dimethyl trisulfide, and 1-Octen-3-Ol showed significantly positive correlations with multiple lipid metabolites such as Lpe (O-20:1), Lpc (O-18:2), Lpe (O-18:0), and 15-Oxo-Ete. This observation indicated that phospholipid hydrolysis and polyunsaturated fatty acid oxidation proceeded during frozen storage. These two processes were tightly coupled to the formation of unpleasant volatile compounds with mushroom-like and sulfurous odors.

Conversely, LPC (20:5-Sn1) and L-proline showed strong negative correlations with 1-octen-3-ol and dimethyl trisulfide. Dominant microorganisms and key lipid metabolites affected the synthesis of key flavor compounds.

## 4. Discussion

Consumers generally consider nutritional quality and umami taste when purchasing seafood. Frozen storage has long been widely used to extend the shelf life of fresh seafood [19]. However, quality deterioration still occurs during frozen storage, encompassing changes in texture, flavor, and water-holding capacity, among others [20,21]. Although the effects of freezing on seafood quality have been investigated for many years, few comprehensive studies have revealed the underlying mechanisms in *P. esculenta*.

Physical indicators including pH, color, WHC, and shear force serve as the most direct basis for evaluating muscle quality [22]. In the present study, the WHC of *P. esculenta* muscle showed a progressive decline over 360 days of frozen storage, with a marked acceleration between day 180 and day 270. This trend is consistent with previous reports in other aquatic species, such as *Portunus trituberculatus* and fish muscle, where prolonged freezing led to reduced WHC due to protein denaturation and damage to muscle fibers [23,24]. However, the relatively moderate decline observed in the later stage (270–360 d) may indicate stronger binding of residual water to the myofibrillar matrix, or that the extent of freeze-induced structural disruption approaches a plateau. This phenomenon possibly indicates that the most labile water fractions are lost during the first half of storage, while the residual water is less susceptible to further release. Collectively, these findings confirm that frozen storage gradually impairs the water-binding properties of *P. esculenta* muscle, with the most critical changes occurring during the mid-term period. The pH value of *P. esculenta* muscle decreased slightly in the first 90 days, dropped significantly by day 180, and remained relatively stable thereafter. Lactic acid generated by the breakdown of muscle glycogen contributes to the observed pH decline [25]. Meanwhile, pH reduction coincides with an increase in solute concentration. Water loss and hydrogen ion release are responsible for this phenomenon; these factors destroy muscle structure and lower the water-holding capacity [26]. However, this pattern differs from some studies on marine crustaceans. During frozen storage, the pH of *Litopenaeus vannamei* exhibited different rising tendencies across all experimental groups [27].

Throughout the 360-day storage period, the TVB-N content in *P. esculenta* muscle increased continuously from 5.77 mg/100 g at day 0 to 15.17 mg/100 g at day 360, and still remained below the 30 mg/100 g limit specified in GB 2733-2015 [18]. Lower TVB-N values reflect weaker degradation of protein-bound tyrosine and methionine. Such limited proteolysis implies a lower degree of nutrient loss. This trend is broadly consistent with observations of frozen crab and shrimp, in which TVB-N accumulation is effectively retarded at sub-zero temperatures [7,28]. The moderate increase observed after 180 days indicates that protein degradation still progresses gradually over extended storage. These results demonstrate that frozen storage is highly effective at retarding the formation of alkaline spoilage products in *P. esculenta*, and thus can maintain the quality over extended storage periods. In this study, TVB-N values in all treatment groups remained below 30 mg/100 g throughout the entire frozen storage period, indicating that all groups remained safe for consumption during this period. TBARS values, reflecting lipid oxidation, exhibited a more pronounced upward trend, particularly after 90 days. This pattern is consistent with previous findings for frozen crustaceans, which indicate that lipid oxidation tends to be more prominent than protein degradation under long-term frozen-storage conditions [29,30]. The accelerated oxidation observed after 90 days can be attributed to progressive damage to cellular membranes caused by ice crystals, which in turn promotes contact between lipids and pro-oxidants [31]. Interestingly, the increase in TBARSs did not directly parallel the trend of TVB-N, indicating that lipid oxidation develops more rapidly than proteolysis under the current frozen storage conditions. This dissociation possibly indicates that the muscle lipid fraction is inherently more susceptible to oxidative deterioration than its protein fraction.

High-throughput sequencing revealed that frozen storage significantly reduced the microbial alpha diversity. This observation is consistent with findings from other seafood [20,32]. Low temperatures inhibit the growth of most microbial populations and select for cold-tolerant or stress-resistant microbial taxa. At the phylum level, the relative abundance of Bacillota rose from 24.46% to 87.52% during frozen storage. This remarkable enrichment indicated that Bacillota exhibited superior cold adaptability compared with other microbial groups during low-temperature preservation. Bacillota are widely reported to possess robust stress resistance, including tolerance to cold-induced osmotic damage, oxidative stress, and freeze–thaw injury [20]. The reduction in the relative abundance of *Pseudomonadota* during frozen storage could be attributed to its poor tolerance to long-term freezing stress. Ice-crystal formation and recrystallization under −20 °C can rupture cell envelopes, trigger intracellular damage and cause DNA degradation in these Gram-negative taxa [33]. These results indicate that long-term frozen storage drives a clear succession of microbial communities, shifting from a Pseudomonadota-dominated structure to a Bacillota-dominated structure, which may have important impacts on flavor development and spoilage characteristics. The enrichment of *Macrococcus* under prolonged freezing conditions suggests this genus has exceptional cold tolerance. LEfSe analysis further supported these shifts, identifying *Macrococcus* and norank *Brocadiales* as biomarkers at 360 d, whereas fresh samples were dominated by marine-associated taxa such as *Paracoccaceae* and *Cyclobacteriaceae*. Collectively, these findings demonstrate that long-term frozen storage fundamentally reshapes the microbial ecology of *P. esculenta* muscle, favoring stress-tolerant Gram-positive taxa and potentially altering spoilage pathways.

The untargeted metabolomics results revealed pronounced metabolic differentiation among fresh, 180 d and 360 d frozen-stored *P. esculenta* muscle samples. Consistent with previous metabolomics findings from frozen aquatic products, purine, nucleotide and arachidonic acid metabolism exhibited significant perturbation during prolonged frozen storage. These metabolisms are commonly regarded as key metabolic signatures reflecting muscle protein catabolism and lipid peroxidation under low-temperature stress [34,35]. Nevertheless, the present study observed a sharp reduction in the number of differential metabolites from 89 (0 d vs. 180 d) to 20 (180 d vs. 360 d). This trend may be explained by the fact that major tissue catabolic events mainly occur within the first 180 days of preservation; after that, muscle biochemical reactions gradually reach a relatively stable state under −20 °C, and only limited metabolic shifts continue. Collectively, these observations indicate that the most dramatic metabolic disturbance of *P. esculenta* muscle takes place within the initial 180-day frozen period, while nucleotide metabolism persistently acts as a core metabolic pathway throughout the whole course of long-term preservation.

Untargeted metabolomics revealed substantial differences in metabolic profiles among fresh, 180 day, and 360 day frozen samples. In total, 89 differential metabolites were identified between the 0-d and 180 d groups, the majority of which were upregulated, including (S)-oleuropeic acid, Gly-Phe-Ile, and p-coumaryl alcohol. This widespread upregulation suggests that early freezing stress triggers a metabolic response characterized by the accumulation of protective osmolytes and antioxidants, which is consistent with findings previously reported in freeze-stressed crustaceans [36]. In contrast, only 45 metabolites were differentially upregulated between the 0-d and 360 d groups, implying that the metabolic shift becomes more focused over time, with fewer but more pronounced changes in specific pathways. KEGG enrichment analysis showed that the most significantly affected pathways were purine metabolism, nucleotide metabolism, arachidonic acid metabolism, and amino acid metabolism. These pathways are involved in energy depletion, protein catabolism and lipid oxidation of aquatic muscle, which reflect the metabolic disturbance triggered by low-temperature frozen stress. The involvement of arachidonic acid metabolism aligns with the observed increase in TBARSs, because arachidonic acid is a primary substrate for lipid oxidation and the generation of volatile oxidation products [29]. Notably, ABC transporters and starch/sucrose metabolism were also enriched in the comparison between 180 d and 360 d groups, suggesting that prolonged frozen storage may alter nutrient transport and carbohydrate utilization in the resident microbiota. These findings indicate that frozen storage not only alters host muscle metabolism but also modulates the metabolic activities of the associated microbial community. Overall, the metabolomic data indicate that both host-derived biochemical reactions and microbial-mediated transformations contribute to the progressive quality deterioration of *P. esculenta* during frozen storage.

The volatile profile of *P. esculenta* muscle shifted markedly during frozen storage, with aldehydes decreasing from 46.86% to 22.19% and alcohols increasing from 20.90% to 38.96% over 360 days. This shift from aldehydes to alcohols aligns with previous findings in frozen fish and crab, showing that prolonged storage promotes the conversion of aldehydes to their corresponding alcohols, a process driven by endogenous alcohol dehydrogenases or microbial activity [37,38]. The aldehydes associated with fresh flavor, such as benzaldehyde and β-ionone, decreased during frozen storage, whereas alcohols including 1-octen-3-ol gradually accumulated. These alterations collectively led to the gradual loss of fresh, floral and fatty flavor attributes, and the emergence of more mushroom-like and earthy notes. OAV analysis further confirmed the dominant role of lipid-oxidation-derived compounds in the overall flavor profile. Notably, (E,E)-2,4-decadienal showed the highest OAV at all detection time points, and its continuous significant increase highlights its dominant contribution to fatty and chicken-fat-like odors. This compound has previously been identified as a key odorant in oxidized seafood [39]. Its sustained increase indicates that lipid oxidation proceeds continuously throughout frozen storage. Conversely, the OAV of (E,E)-2,4-nonadienal, which imparts desirable fatty and fruity notes, decreased after 180 days, reflecting the gradual loss of pleasant aroma attributes. The most notable off-flavor markers were 2-pentylfuran, dimethyl disulfide, and dimethyl trisulfide, all of which showed substantial OAV increases during storage. The sharp increase in dimethyl trisulfide is particularly notable, strongly indicating that sulfur-containing volatiles produced from the microbial catabolism of sulfur-containing amino acids become increasingly dominant over the storage period. This pattern may be explained by the combined effects of lipid autoxidation and microbial enzymatic activities. In summary, the OAV data confirm that the flavor deterioration of *P. esculenta* during frozen storage is primarily driven by the gradual accumulation of oxidative and sulfurous off-odors, accompanied by the loss of fresh and fruity flavor characteristics.

Correlation analysis revealed multiple strong associations between physicochemical indicators, microbial taxa, and volatile compounds, providing mechanistic insights into the flavor deterioration process. TBARSs showed significant positive correlations with 1-octen-3-ol, dimethyl trisulfide, and dimethyl disulfide, reinforcing the link between lipid oxidation and the formation of key off-odor volatiles. This is in good agreement with established knowledge that lipid-derived aldehydes and furans undergo further transformations to yield sulfurous and alcohol compounds under oxidative conditions [40]. *Macrococcus* displayed strong positive correlations with dimethyl trisulfide, 1-octen-3-ol, and 3-methyl-1-pentanol, implying that this cold-adapted genus may contribute to the production of these off-flavors, possibly via proteolytic and lipolytic activities. In contrast, unclassified *Paracoccaceae* and norank *Cyclobacteriaceae* were negatively correlated with these volatile compounds. This indicates that these taxa associated with fresh products may compete with spoilage microorganisms, or lack the enzymatic systems required to generate these off-flavor compounds.

The observed correlations between key lipid metabolites and off-odor volatiles further indicate that phospholipid hydrolysis and polyunsaturated fatty acid oxidation are closely associated with flavor deterioration. For instance, L-proline was negatively correlated with sulfurous compounds. This indicates that free amino acids are consumed or transformed over prolonged storage, thereby changing the pool of precursors for volatile synthesis. Collectively, these integrated correlation analyses demonstrate that quality and flavor deterioration of *P. esculenta* muscle arises from the interplay among lipid oxidation, protein degradation and microbial succession.

## 5. Conclusions

During 360-day frozen storage, *P. esculenta* muscle showed declining water-holding capacity, slight pH drop, and rising TVB-N/TBARS content. Microbial diversity decreased, with a community shift from Pseudomonadota to Bacillota, dominated by cold-tolerant Macrococcus. Untargeted metabolomics identified 4299 metabolites; differential ones linked to purine, nucleotide, arachidonic acid, and amino acid metabolism. Volatile profiles changed markedly: fresh aldehydes decreased, whereas off-flavor alcohols (1-octen-3-ol) and sulfur-containing (dimethyl trisulfide) volatile compounds continuously increased. Pearson correlation linked spoilage indicators, cold-adapted microbes, and lipid metabolites to off-odor formation. This multi-omics approach clarified deterioration mechanisms and supports future optimization of frozen storage strategies. Future work could validate the discriminatory potential of these key volatile metabolites and *Macrococcus* as spoilage biomarkers for rapid quality monitoring of frozen *P. esculenta*.

Furthermore, targeted interventions, including optimized pre-freezing treatments, could be tested to suppress cold-tolerant microbial metabolism and lipid oxidation, thereby retarding off-flavor development during long-term frozen storage.

## Figures and Tables

**Table 1:** Alpha diversity of the microbiome of *P. esculenta* muscle during frozen storage.

Frozen Storage Time (Days)	Chao1	Ace	Shannon	Simpson
0	545.38 + 86.69 ^a^	545.36 + 86.36 ^a^	4.33 + 0.33 ^a^	0.04 + 0.01 ^a^
180	389 + 34.04 ^b^	389.06 + 34.12 ^b^	4.02 + 0.05 ^a^	0.04 + 0.00 ^a^
360	386.68 + 38.69 ^b^	386.55 + 38.58 ^b^	3.19 + 0.38 ^b^	0.17 + 0.07 ^b^

Note: Different lowercase letters in the same column indicate significant differences (*p* < 0.05) during frozen storage times.

## Data Availability

The original contributions presented in the study are included in the article/Supplementary Material. Further inquiries can be directed to the corresponding author.

## Supplementary Materials

The following supporting information can be downloaded at: https://www.mdpi.com/article/10.3390/biology15191789/s1, Figure S1: PCA score plot of each sample at different frozen storage time points; Table S1: Statistics of sequencing data for each sample during frozen storage; Table S2: Chemical classification statistics of metabolites; Table S3: Statistics of relative contents of volatile substance.

## Abbreviations

The following abbreviations are used in this manuscript:

WHC	Water-holding capacity
TVB-N	Total volatile basic nitrogen
TBARSs	Thiobarbituric acid reactive substances
VOCs	Volatile organic compounds
MDA	Malondialdehyde
HS-SPME-GC-MS	Headspace solid phase microextraction coupled with gas chromatography-mass spectrometry
LC-MS	Liquid chromatography-mass spectrometry
