# Supplementary material for: Changes in Physicochemical Properties, Volatile Flavor Compounds and Microbiota in Phascolosoma esculenta Muscle During Frozen Storage at −20 °C
Source: Biology (Basel). 2026 Oct 8;15(19):1789. doi: 10.3390/biology15191789 (PMC13664345; doi:10.3390/biology15191789)
Supplement: Supplementary file 1 [file biology-15-01789-s001.zip › Figure S1. PCA score plot of each sample at different frozen storage time points.pdf]

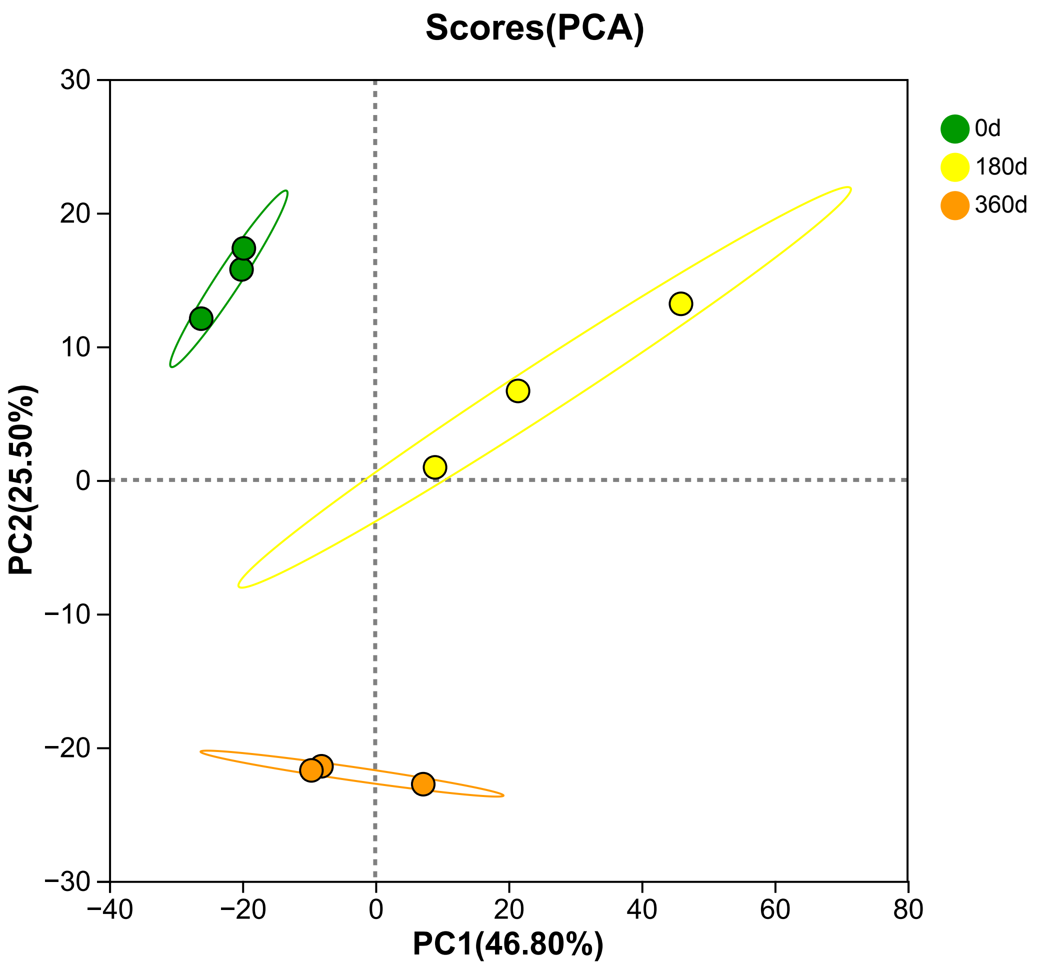
Scores(PCA)
PC2(25.50%)
PC1(46.80%)
30
20
10
0
−10
−20
−30
−40
−20
0
20
40
60
80
0d
180d
360d
