# Supplementary material for: Changes in Physicochemical Properties, Volatile Flavor Compounds and Microbiota in Phascolosoma esculenta Muscle During Frozen Storage at −20 °C
Source: Biology (Basel). 2026 Oct 8;15(19):1789. doi: 10.3390/biology15191789 (PMC13664345; doi:10.3390/biology15191789)
Supplement: Supplementary file 1 [file biology-15-01789-s001.zip › Table S1 Statistics of sequencing data for each sample during frozen storage.pdf]

Table S1. Statistics of sequencing data for each sample during frozen storage.

Sample_info	Seq_num	Base_num(bp)	Mean_length(bp)	Min_length(bp)	Max_length(bp)
0d-1	116102	48154391	414.75936	228	523
0d-2	107110	43921886	410.06336	229	448
0d-3	117170	48320510	412.3966	232	518
180d-1	84890	35236209	415.0808	249	432
180d-2	88198	36653009	415.57642	263	485
180d-3	103916	43036750	414.14941	323	443
360d-1	113425	48204784	424.99259	250	540
360d-2	109062	46116119	422.84314	262	540
360d-3	129694	54909303	423.37582	230	540
